# Evaluation of the impact of polyclonal infection and heteroresistance on treatment of tuberculosis patients

**DOI:** 10.1038/srep41410

**Published:** 2017-01-25

**Authors:** Mansour Kargarpour Kamakoli, Hamid Reza Sadegh, Ghazaleh Farmanfarmaei, Morteza Masoumi, Abolfazl Fateh, Gholamreza Javadi, Fatemeh Rahimi Jamnani, Farzam Vaziri, Seyed Davar Siadat

**Affiliations:** 1 Department of Biology, Science and Research Branch, Islamic Azad University, Tehran, Iran; 2 Department of Mycobacteriology and Pulmonary Research, Pasteur Institute of Iran, Tehran, Iran; 3 Microbiology Research Center (MRC), Pasteur Institute of Iran, Tehran, Iran

## Abstract

Mixed strain infections of *Mycobacterium tuberculosis* make diagnosis, treatment, and control of tuberculosis (TB) more difficult. This study was aimed to evaluate the relationship between mixed infections, antibiotic resistance patterns and treatment of TB patients. In this study, among 2850 suspected TB clinical samples, a total of ninety-six clinical samples from 66 TB confirmed patients were subjected to the 24-locus variable-number tandem repeat method to evaluate the prevalence of mixed infections. For all studied strains, 288 colonies (three individual clones for each sample) were isolated from different colonies and separately analyzed by the Drug Susceptibility Test (DST). For all patients, follow up was done after 6 months of treatment. Based on direct 24 loci MIRU-VNTR, in the 66 TB patients, 53% (35/66) showed mixed infection. In the mixed samples, 45.71% (16/35) showed different antibiotic resistant patterns. Among the mixed infection patients, eight (22.9%; 8/35) showed treatment failure after six- month therapy. Six of these non-treated patients (75%; 6/8) had different antibiotic resistant patterns. We conclude that mixed infections, have a negative impact on treatment of TB patients especially when co-infecting *M. tuberculosis* strains display heteroresistance.

Nowadays, molecular genotyping methods can detect the phenomenon of mixed (polyclonal) infections in tuberculosis (TB). In mixed infections multiple strains of *Mycobacterium tuberculosis (M. tuberculosis*) are retrieved from an individual patient^1^. Mycobacterial Interspersed Repetitive Unit- Variable Number Tandem Repeat (MIRU-VNTR) genotyping, widely applied in the molecular epidemiology of TB, eases the detection of mixed infections^2^. Based on MIRU-VNTR, mixed infections are defined by the presence of different MIRU-VNTR patterns at two or more loci in the same clinical samples, while clonal heterogeneity is defined as having different patterns at a single locus^3^,^4^.

There are several studies in which mixed *M. tuberculosis* infections were evaluated in various geographic areas, but there have been few efforts to examine the impact of this phenomenon on treatment of TB patients^5^. In association with polyclonal infections, another important issue is hetero-resistance which is defined as the coexistence of susceptible and resistant strains in the same clinical sample^6^.

This study was aimed to evaluate the impacts of mixed infections and heteroresistance on treatment of TB patients.

## Results

### Detection of mixed infections

Based on direct 24 loci MIRU-VNTR, in the 66 TB patients, 53% (35/66) showed mixed infection (Supplementary File 1). Among the MIRU-VNTR loci, those of 2401, 577, 154, 2996 and 960 were more successful in the identification of mixed infections.

### Detection of heteroresistance

A total of 288 colonies (three individual clones for each sample) were analyzed for DST. Among the 66 TB patients, 24.24% (16/66) showed heteroresistance. In mixed samples 45.71% (16/35) showed different resistance patterns (P = 0.030). None of the single samples showed heteroresistance (Supplementary File 1).

### Follow up

Among the mixed infection patients, eight (22.9%, 8/35) showed treatment failure after therapy for six months (*P* = 0.001). Six of these non-treated patients (75%; 6/8) had different antibiotic resistant patterns (Supplementary File 1). Although there was no significant association between the type of resistance pattern and therapy failure (*P* > 0.05), there was a strong significant association between heteroresistance and treatment failure (*P* = 0.0001).

## Discussion

Although the existence of mixed *M. tuberculosis* infections is now generally accepted^7^,^8^,^9^, comprehensive studies of their impact on treatment are rare. In our study we evaluated this issue among 66 confirmed TB patients. We showed that 53% of the patients showed mixed infection based on direct genotyping of the clinical samples. This demonstrated the high prevalence of polyclonal infection in these TB patients. In comparison with our previous study in which genotyping was performed on cultures, the current study demonstrates that direct genotyping better reveals the polyclonal infection^10^. The strength of direct genotyping was that the results were not limited only to patients with positive smears and cultures.

Polyclonal *M. tuberculosis* infections may have a negative impact on drug resistance testing performed by both phenotypic (*e.g.,* proportional test) and genotypic methods (*e.g.,* GeneXpert MTB/RIF)^11^,^12^. Another problem for diagnosis and treatment of TB patients, which is more difficult to detect by the usual phenotypic DST is hetero-resistance^13^. Accordingly, we decided to perform DST on three single colonies for each clinical sample. Among the 35 patients with mixed infections, 16 (45.71%) showed heteroresistance. Among these 16 patients, six were detected as non-treated patients after six-month follow-up. There was a meaningful association between patients infected with mixed strains, heteroresistance and treatment failure (*P* < 0.05).

Our results are in agreement with a previous study which have demonstrated that infections with heteroresistance can compromise treatment outcomes^14^. In contrast, Cohen *et al*. showed that the negative effect of polyclonal infections on the response to early treatment appeared to be independent of a relationship with drug resistance in TB patients in KwaZulu-Natal, South Africa^15^. This difference may be related to the follow-up period; which ended at six and two months in our and Cohen’s study, respectively.

There was no significant association between the type of antibiotic resistance pattern and treatment failure in our study. Interestingly, we showed that in one patient among the untreated patients (Patient number 45) with a history of antibiotic therapy, treatment conferred a competitive growth advantage to a multidrug-resistant *M. tuberculosis* strain. This result is supported by previous reports^16^,^17^.

Thus, the mixed-infection patients with heteroresistance represents a risk group which may contribute to an increased risk of treatment failure. Therefore, early identification of patients with mixed infections would enable prompt therapy with an optimized drug regimen increasing the cure rate^18^. High resolution whole genome sequencing analysis will elucidate more details in this regard^19^. Mixed-infections in TB can be ruled out only by using molecular techniques (e.g. MIRU-VNTR) of pre-culture sample and analysis of multiple colonies. Prompt detection of polyclonal infections with distinct drug susceptibility patterns will enable suitable drug regimens, which could prevent treatment failure.

We conclude that mixed infections, have a negative impact on treatment of TB patients when co-infecting *M. tuberculosis* strains display heteroresistance.

## Methods

### Sample collection

This study was conducted between January 2015 and January 2016. In this period, we received 2850 suspected TB clinical samples. After excluding the non tuberculosis mycobacteria and *M. bovis,* a total of 66 TB patients were confirmed, who were mainly from Tehran province. Most of the clinical samples in our study were sputa, with one pleural fluid, one axillary abscess and two gastric juice samples. Clinical and demographics data were obtained. All experiments were performed in accordance with guidelines approved by Pasteur Institute International networks. Ethical reviews and informed consent approval were granted by the Ethical Committee of the Pasteur Institute of Iran. Informed consent was obtained from all patients in this study. The results of this research did not influence TB patients’ treatment.

### Detection of mixed infection

#### DNA extraction

Genomic DNA was extracted from clinical samples with the Proba-NK DNA extraction kit (DNA-Technology Company, Moscow, Russia) according to the manufacturer’s instructions. The DNA was stored at −20 °C until used for molecular studies.

#### MIRU-VNTR genotyping

For screening of mixed infection in our TB patients, MIRU-VNTR was performed on clinical samples which elucidate the polyclonal infection in the whole population of bacteria. Manual MIRU-VNTR genotyping was performed as described by Supply *et al*.^20^. For each locus, deionized water and DNA from *M. tuberculosis* H37Rv were used as a negative and positive control, respectively. Amplicons were electrophoretically separated on 1.8% agarose gels, using a 100-bp DNA ladder as size markers. Polyclonal infection was defined to be present if a single sample had more than one amplicon at two or more loci. To avoid laboratory cross contamination, all standard precautions were observed. In addition, absence of amplicon and presence of double alleles were confirmed by three independent round sets of PCRsd.

#### Culture and DST

Ninety-six clinical specimens from 66 TB patients were processed using standard methods and inoculated in Lowenstein-Jensen (LJ) medium. For all patients DST was done on three single colonies for each clinical sample (Based on the availability of samples, for nine patients three sputa, for twelve patients two sputa and for remaining patients one clinical sample were analyzed). DST was done using the proportion method according to the WHO guidelines based on the following antibiotic concentrations: rifampin (RIF; 40.0 μg/ml), isoniazid (INH; 0.2 μg/ml), streptomycin (STR; 4 μg/ml) and ethambutol (EMB; 2.0 μg/ml). Bacterial growth on antibiotic-containing media exceeding 1% of the number of colonies on antibiotic-free media were considered to be resistant to the antibiotic. There were two sets of DST readings, the first at 28 days and a further reading after 42 days of incubation.

#### Patients follow up

Double blind follow up of TB patients was carried out by the physician during the period of six-month treatment (based on WHO recommendation; two months of intensive phase and four months of continuation phase). After this period, the clinical (chest X-ray) and microbiological diagnostic procedures (microscopy and culture of clinical sample) were carried out to evaluate the success or failure of treatment.

### Statistical analysis

Multivariable logistic regression was used. All calculations were performed using SPSS version twenty-two (SPSS Inc., Chicago, IL, USA). A *p* value of <0.05 was considered significant.

## Additional Information

**How to cite this article:** Kargarpour Kamakoli, M. *et al*. Evaluation of the impact of polyclonal infection and heteroresistance on treatment of tuberculosis patients. *Sci. Rep.* **7**, 41410; doi: 10.1038/srep41410 (2017).

**Publisher's note:** Springer Nature remains neutral with regard to jurisdictional claims in published maps and institutional affiliations.

## Supplementary Material

Supplementary Information
