# Supplementary material for: Evaluation of the impact of polyclonal infection and heteroresistance on treatment of tuberculosis patients
Source: Sci Rep. 2017 Jan 25;7:41410. doi: 10.1038/srep41410 (PMC5264600; doi:10.1038/srep41410)
Supplement: Supplementary Information [file srep41410-s1.pdf]

Evaluation of the impact of polyclonal infection and heteroresistance on treatment of tuberculosis patients

Mansour Kargarpour Kamakoli, Hamid Reza Sadegh, Ghazaleh Farmanfarmaei, Morteza Masoumi, Abolfazl Fateh, Gholamreza Javadi, Fatemeh Rahimi Jamnani, Farzam Vaziri and Seyed Davar Siadat

Supplementary 1. Results of MIRU-VNTR, DST and treatment failure of 66 TB patients.

Patient	ID NUMBER	MICROSCOPY	MIRU-VNTR loci																								INH	SM	RMP	EMB	Treatment failure
			154	424	577	580	802	960	1644	1955	2059	2163b	2165	2347	2401	2461	2531	2687	2996	3007	3171	3192	3690	4052	4156	4348					
1	1642	3+	2+3	2	2+4	2	1	2+3	3	na	na	13	3	4	2+9	2	6	1	5+8	3	3	3	5	5	na	2	S	S	S	**S**	YES
																											S	S	S	**R**	
																											S	S	S	**R**	
	1643	1+	2+3	2	2+4	2	1	2+3	3	na	na	13	3	4	2+9	2	6	na	5+8	na	3	3	na	na	na	2	S	**S**	S	S	
																											S	**R**	S	S	
																											S	**R**	S	S	
	1644	1+	2+3	2	2+4	2	1	2+3	3	na	na	13	3	4	2+9	2	6+13	na	5+8	3	3	3	na	na	na	2	S	S	S	S	
																											S	S	S	S	
																											S	S	S	S	
2	1877	1+	2+3	2+4	2	2	1	1+6	4	na	na	na	4	4	2+9	2	5	1+3	8	na	3	3	na	na	na	3	**S**	S	S	S	NO
																											**R**	S	S	S	
																											**R**	S	S	S	
	1887	8AFB	2+3	2	2	2	14	na	3	na	na	13	na	4	2+9	1+2	na	1+3	9	na	2+3	3	na	na	na	na	S	S	S	S	
																											S	S	S	S	
																											S	S	S	S	
	1888	1+	4	na	2	2	na	na	3	na	na	na	4	4	10	2	5	9	6	2	na	3	na	na	na	5	S	S	S	S	
																											S	S	S	S	
																											S	S	S	S	
3	54	3+	2	na	2	2	3	5	na	na	4	na	na	4	2	3	11	na	8	3	na	3	3	8	na	3	S	S	R	S	NO
																											S	S	R	S	
																											S	S	R	S	
	55	2+	2	na	2	2	na	5	4	na	2	na	na	4	2+9	2	5	1	8	3	3	3	3	na	na	3	S	S	S	S	
																											S	S	S	S	
																											S	S	S	S	
	56	4+	2+4	1	2	2	3	5	4	na	6	na	na	4	2+9	2	5	1	8+9	3	3	3	3	3+8	na	3	S	S	S	S	
																											S	S	S	S	
																											S	S	S	S	
4	4037	2+	2+4	1+4	2	2	3	7	4	na	na	1+2	2	4	4+9	3	5	1	9	3	3	3	3	4	na	3	R	R	S	S	NO
																											R	R	S	S	
																											R	R	S	S	
	4063	1+	2	1	4+6	2	2+3	2+3	3	na	na	3	4+5	2+4	2+4	2+6	4+5	1+2	5	3	3	3	2+4	na	10	2+3	R	R	S	**S**	
																											R	R	S	**R**	
																											R	R	S	**R**	
	4081	2+	2	0+4	4+6	2	2+3	2+3	3	na	na	3	4+5	2+4	2+4	2+6	4+5	2	5	1+3	3	3	2+4	15	10	2+3	R	R	S	**S**	
																											R	R	S	**R**	
																											R	R	S	**R**	

**5**	4412	3+	2	2	4	2	2	3	3	na	2	3	2	4	2	3	6	1	5	3	3	3	3+6	5	3	2	S	S	R	S	YES
																											S	S	R	S	
																											S	S	R	S	
	4426	3+	2	2	4	2	4	2	2	na	na	1+2	3	4	2	1	5	1	5	3	2	3	3	5	na	2	S	S	R	S	
																											S	S	R	S	
																											S	S	R	S	
	4455	3+	2	3	4	2	4	na	na	na	na	1+2	3	na	2	1	5	1	5	2+3	2	3	3	5	na	2	S	S	R	**S**	
																											S	S	R	**R**	
																											S	S	R	**R**	
**6**	1608	1+	na	2	2+3	na	na	4	na	8+13	na	6	na	5	2+8	3	3	na	7+12	2	3	3	na	na	na	na	**S**	S	S	**R**	NO
																											**R**	S	S	**S**	
																											**R**	S	S	**S**	
	1618	8AFB	na	2	2+3	na	na	4	na	na	na	10	3	na	2	na	na	na	8	2+0	3	3	na	na	5+9	0	**S**	S	S	**R**	
																											**R**	S	S	**S**	
																											**R**	S	S	**S**	
	1629	1+	na	1	2+3	na	na	4	na	na	na	3+11	na	na	2	3	na	na	3	1	3	3	na	na	8+13	0	**S**	S	S	**R**	
																											**R**	S	S	**S**	
																											**R**	S	S	**S**	
**7**	1058	1+	2	4	2	3	4	9	5	2	2	na	4	6	4+8	2	2	2	7+9	3	4	3	na	na	2	3	S	S	S	R	NO
																											S	S	S	R	
																											S	S	S	R	
	1070	2+	2	na	2	3	4	9	5	2	na	na	4	5	4	2	2	2	7+9	2	4	3	na	na	na	3	S	S	S	R	
																											S	S	S	R	
																											S	S	S	R	
	1071	1+	2	4	na	3	4+9	9	na	1	2	na	4	6	4	2	2	2	7+9	na	4	3	na	na	na	3	S	S	S	R	
																											S	S	S	R	
																											S	S	S	R	
**8**	2	3+	2	2	5	2	3	2	2	na	2+6	2	2	4	2+5+9	2	10	1	8+9	3	3	3	3	3+6	na	2	S	S	S	S	NO
																											S	S	S	S	
																											S	S	S	S	
	8	2+	2	1	2+4	2	3	2	3	na	na	0	2	4	2	1	5	1	7	3	2	3	3	7	na	2	S	S	S	S	
																											S	S	S	S	
																											S	S	S	S	
	9	1+	2+4	2	3+4	2	3	2	2	na	na	na	2	4	2+5+9	1	10	na	8+9	3	3	3	3	3+8	na	2	S	S	S	S	
																											S	S	S	S	
																											S	S	S	S	
**9**	4169	1+	3	na	na	3	1	na	na	na	na	na	3	na	1	na	na	na	na	3	2	3	3	na	na	na	S	S	R	S	NO
																											S	S	R	S	
																											S	S	R	S	
	4215	1+	2	0	5	3	1	3	3	1+2	na	3	3	3	1	3	6	5	2	3	2	3	3	5	na	1	S	S	R	S	
																											S	S	R	S	
																											S	S	R	S	
	4228	1+	na	na	na	3	1	na	na	1	na	na	na	na	1+4	3	na	1	na	na	2	3	na	na	na	3	S	S	R	S	
																											S	S	R	S	
																											S	S	R	S	

**10**	2200	1+	2+4	2	4	2	na	8	na	na	na	na	3+6	na	na	3	na	na	11+12	na	na	3	3	6+8	na	na	S<br>S<br>S	S<br>S<br>S	S<br>S<br>S	S<br>S<br>S	YES
	2231	1+	1	2	3+4	2+4	3	2	2	na	na	na	3+6	4	2+9	2	9	0	4+13	1	4	3	3	na	na	2	S<br>S<br>S	S<br>S<br>S	**S**<br>**R**<br>**R**	S<br>S<br>S	
**11**	1580	NEG	2+4	3	2+4	2	na	3	3	na	na	na	na	na	2	2	5+6	1	5+6	3	3	3	3+5	na	na	2	S<br>S<br>S	S<br>S<br>S	S<br>S<br>S	S<br>S<br>S	YES
	1613	NEG	2	3	2+4	2	na	na	4	na	na	10+14	na	na	10	3	5	1	6	na	3	3	na	na	na	0+2	S<br>S<br>S	S<br>S<br>S	S<br>S<br>S	S<br>S<br>S	
**12**	1113	4+	2	4	2	3	4	10	4	3	2	na	2	5	4	3	6	2	9	3	4	3	na	5	2	3	R<br>R<br>R	R<br>R<br>R	S<br>S<br>S	S<br>S<br>S	NO
	1114	4+	2	4	2	3	4	10	4	3	2	na	2	5	4	3	6	2	9	3	4	3	na	5	na	3	R<br>R<br>R	R<br>R<br>R	S<br>S<br>S	S<br>S<br>S	
**13**	1607	2+	2	4	2	2	3	7	na	na	na	2	2	4	2+4+9	2	5	1	9	3	3	3	3	na	na	3	S<br>S<br>S	R<br>R<br>R	S<br>S<br>S	S<br>S<br>S	NO
	1660	3+	2+3	4	2	2	3	6	4	na	na	na	2	4	4	2	5	1	9	3	na	3	3	4	na	3	S<br>S<br>S	R<br>R<br>R	S<br>S<br>S	S<br>S<br>S	
**14**	1700	1+	na	4	3	2	na	na	na	8	na	na	3	na	4	3	6	1+3	9	3	3	3	3	na	8	na	R<br>R<br>R	R<br>R<br>R	S<br>S<br>S	S<br>S<br>S	YES
	1747	NEG	2+4	1+4	2	2	2+3	na	4	na	na	na	2+9	4+10	4+9	2	5	1+3	9	3	3	3	3	na	8	3	R<br>R<br>R	R<br>R<br>R	S<br>S<br>S	S<br>S<br>S	
**15**	2038	2+	2+13	2+4	2	2	1+4	2+5	4	na	na	na	4	4	2+9	2	5+6	1	na	3	3	3	3	na	na	4	S<br>S<br>S	**S**<br>**R**<br>**R**	R<br>R<br>R	S<br>S<br>S	NO
	2056	2+	2	2	2	2	4	5	4	na	2+5	na	4	4	2+8	2	5	1	5+6	2	4	3	3	8	na	6	S<br>S<br>S	**S**<br>**R**<br>**R**	R<br>R<br>R	S<br>S<br>S	
**16**	1704	NEG	2+4	na	2	2	na	na	3	na	na	na	na	na	10	na	5	na	6	na	3	3	na	na	na	2	S<br>S<br>S	S<br>S<br>S	S<br>S<br>S	S<br>S<br>S	NO
	1714	NEG	2	3+8	2+4	2	3	3+5	3	na	na	na	3	na	2+10	2	6	1	5+6	3	3	3	5	3	na	2	S<br>S<br>S	S<br>S<br>S	S<br>S<br>S	S<br>S<br>S	
**17**	1786	3+	2	3	2+4	2	3	3	3	na	2	14	3	4	2+10	1	5	1	5+6	3	3	3	3	na	na	0+2	S<br>S<br>S	S<br>S<br>S	S<br>S<br>S	S<br>S<br>S	NO

	1788	2+	na	3	2+4	2	3	2+9	3	na	na	10+14	3	4	2+10	1	5	1+3	5+6	na	2	3	3	na	na	0+2	S<br>S<br>S	S<br>S<br>S	S<br>S<br>S	S<br>S<br>S	
**18**	1157	3+	0+2	2	3+11	3	3	0+2	2	6	2	13	4	5	4+8	3	6	2	9	1	0+7	3	na	2	na	3	R<br>R<br>R	R<br>R<br>R	S<br>S<br>S	R<br>R<br>R	NO
	1158	3+	2	3	3	3	3	2	2	6	2	na	4	5	4+8	3	6+9	2	7	1	4	3	na	2+5	na	3	R<br>R<br>R	**R**<br>**S**<br>**S**	S<br>S<br>S	R<br>R<br>R	
**19**	1178	1+	2	4	3	3	3	9	2	3	2	na	4	5	6	2	na	2	na	2	4	3	na	na	na	3	S<br>S<br>S	S<br>S<br>S	S<br>S<br>S	R<br>R<br>R	NO
	1187	2+	2	4	3	3	3	10	2	3	2	na	4	5	4	3	6	2	11	2	3	3	na	na	7	3	S<br>S<br>S	S<br>S<br>S	S<br>S<br>S	R<br>R<br>R	
**20**	779	2+	2	2	2	2	3	8	4	na	2	2	3	4	2	2	5	1	5	3	3	3	3	9	8	3	S<br>S<br>S	S<br>S<br>S	S<br>S<br>S	S<br>S<br>S	NO
	796	NEG	na	na	na	na	na	na	na	na	na	6	na	na	na	na	na	na	na	na	3	3	na	na	2	na	S<br>S<br>S	S<br>S<br>S	S<br>S<br>S	S<br>S<br>S	
**21**	4472	1+	2	10	na	3	1	3	3	1	na	na	3+5	na	na	3	6	1	na	3	1	3	3	5	na	1	S<br>S<br>S	S<br>S<br>S	R<br>R<br>R	S<br>S<br>S	NO
	4542	1+	2	na	na	3	1+2	na	3	na	na	na	6	na	1+4	3	4	2	3	na	2	3	1+4	2	9	2	S<br>S<br>S	S<br>S<br>S	R<br>R<br>R	**S**<br>**R**<br>**R**	
**22**	1670	1+	2	5	2	2	3	6	na	4	1+2	2	4	4	2	2	5	1	8	3	3	3	3	8	na	3	S<br>S<br>S	R<br>R<br>R	S<br>S<br>S	S<br>S<br>S	NO
**23**	375	1+	2	2	2+4	2	3	2+3	3	na	na	14	4	4	2+5	1	5	1+10	5	3	2+3	3	na	na	8	2	S<br>S<br>S	**R**<br>**S**<br>**S**	S<br>S<br>S	S<br>S<br>S	NO
**24**	625	3+	2	2	4	2	3	3	3	11	na	2	3	4	2	1	5	1	5	3	2	3	2	7	na	2	S<br>S<br>S	S<br>S<br>S	S<br>S<br>S	S<br>S<br>S	NO
**25**	960	1+	2	2	4+5	2	7	2+3	3	na	4	14	3+4	4	2+5+8	1	5	1	5	3	2+3	3	na	na	8	2	**S**<br>**R**<br>**R**	R<br>R<br>R	S<br>S<br>S	S<br>S<br>S	YES
**26**	502	2+	2+3	1+2	2+4	2	4	2	3	na	na	14	3	4	2+5+8	1+8	5	1	5+8	3	2+3	3	4	na	8	2	S<br>S<br>S	S<br>S<br>S	S<br>S<br>S	S<br>S<br>S	NO
**27**	629	3+	2	2	2+4	2	3	3	3	na	1	2	2	4	2+5+8	1	5	1	2+6	3	2	3	3	2+7	na	2	S<br>S<br>S	R<br>R<br>R	S<br>S<br>S	S<br>S<br>S	NO

**28**	559	NEG	2+10	na	4	2	1+4	2	3	2+5	na	2	4	na	2	na	5	1+10	2	3	3	3	na	5	8	2	S<br>S<br>S	S<br>S<br>S	R<br>R<br>R	R<br>R<br>R	NO
**29**	3582	3+	2+7	2	4	2	1+3	na	na	2+5	na	2	3	4	2	1	6	1	6+7	3	2	3	na	na	na	2	S<br>S<br>S	R<br>R<br>R	R<br>R<br>R	**S**<br>**R**<br>**R**	NO
**30**	1661	1+	2+3	2	2+4	2	1	na	3	na	10	13	3	na	2+9	1+2	na	na	na	3	2	3	na	na	na	2	R<br>R<br>R	R<br>R<br>R	S<br>S<br>S	**S**<br>**R**<br>**R**	NO
**31**	1804	9AFB	2+3	2	2	2	na	1	3	na	7	na	3	4	9	1	5	1+3	9	3	2	3	na	na	na	na	S<br>S<br>S	S<br>S<br>S	S<br>S<br>S	S<br>S<br>S	NO
**32**	1965	3+	2	1	5	2	2	3	3	na	na	13	4	4	2	2	3	1	5	3	2	3	4	7	na	2	S<br>S<br>S	S<br>S<br>S	S<br>S<br>S	S<br>S<br>S	NO
**33**	2652	1+	2	4	2	2	3	7	4	4	2	na	2	4	4+8	2	5	1	10	3	3	3	3	na	na	3	S<br>S<br>S	R<br>R<br>R	S<br>S<br>S	S<br>S<br>S	NO
**34**	2509	3+	2	2	4	2	3	2	3	5	2	na	3	4	2	1	5	1	5	3	2	3	3	na	na	2	S<br>S<br>S	S<br>S<br>S	R<br>R<br>R	S<br>S<br>S	NO
**35**	2002	NEG	2+14	4	2+4	2	3	na	2	na	na	13	4	4	4+9	2	5	1+3	na	na	3	3	3	na	na	3	S<br>S<br>S	S<br>S<br>S	S<br>S<br>S	S<br>S<br>S	NO
**36**	1339	NEG	2	3	2+4	2	na	na	3	na	2	na	3	na	na	2	5	1	6	na	3	3	na	na	na	2	S<br>S<br>S	S<br>S<br>S	S<br>S<br>S	S<br>S<br>S	NO
**37**	1129	NEG	2	3	2	2	1	5	3	na	na	na	na	na	na	na	5+6	1	5	9	3	3	1	na	na	2	S<br>S<br>S	S<br>S<br>S	S<br>S<br>S	R<br>R<br>R	NO
**38**	2046	3+	2+4	3	2	2	na	5	4	na	na	14	na	na	2	2	5	1	6	na	3	3	3	na	na	5	S<br>S<br>S	S<br>S<br>S	S<br>S<br>S	S<br>S<br>S	NO
**39**	1179	3+	2	4	2	2	3	7	4	na	1	14	3	4	2	2	5	1	5+6	3	3	3	3	na	na	5	S<br>S<br>S	S<br>S<br>S	S<br>S<br>S	S<br>S<br>S	NO
**40**	1871	NEG	5	2	2+5	2	3	4	3	na	na	na	na	4	2+4	2	5	1	5	3	3	3	3+5	na	na	2	S<br>S<br>S	S<br>S<br>S	S<br>S<br>S	S<br>S<br>S	NO
**41**	2016	NEG	na	1+2	2+3	2	na	na	4	na	na	na	14	na	2+10	3	6+9	na	na	na	na	3	na	9	na	2+3	R<br>R<br>R	R<br>R<br>R	R<br>R<br>R	**S**<br>**R**<br>**R**	YES
**42**	2028	NEG	na	na	2+7	na	na	na	4	na	na	14	na	na	10	2+11	6	na	5+9	na	3	3	2+3	na	na	na	S<br>S<br>S	S<br>S<br>S	S<br>S<br>S	S<br>S<br>S	NO

**43**	1748	NEG	2	2	2+4	2	3	2	2	na	na	na	3	3	2	1	5	1	5	3	2	3	3	7	na	2	S<br>S<br>S	S<br>S<br>S	S<br>S<br>S	S<br>S<br>S	NO
**44**	1926	1+	na	2	2+3	2	na	na	na	na	2	1+2	4	4	na	na	5	na	na	7+14	3	3	3+5	2+6	na	na	**S**<br>**R**<br>**R**	R<br>R<br>R	S<br>S<br>S	S<br>S<br>S	NO
**45**	4562	2+	2	0+4	4+6	2	2+3	2+3	3	na	na	3	4+5	2+4	2+4	2+6	4+5	1+2	5	1+3	3	3	2+4	15	10	3	**S**<br>**R**<br>**R**	R<br>R<br>R	R<br>R<br>R	R<br>R<br>R	YES
**46**	2057	NEG	2	na	na	2	4	na	na	na	na	2	na	4	2	1+2	5	na	na	3	na	3	3	na	na	2	S<br>S<br>S	S<br>S<br>S	S<br>S<br>S	S<br>S<br>S	NO
**47**	3157	3+	2	2	4	2	3	2	3	na	na	2	3	3	2	1	5	1	5	3	2	3	3	7	na	1	S<br>S<br>S	S<br>S<br>S	R<br>R<br>R	S<br>S<br>S	NO
**48**	622	3+	2	2	5	2	6	3	5	5	na	2	4	na	3+5+8	1	6	1	6	3	2	3	3	10	9	3	S<br>S<br>S	S<br>S<br>S	R<br>R<br>R	S<br>S<br>S	NO
**49**	273	2+	2	5	2	2	4	9	6	4	na	1	5	4	3	1+2	5	1	8	3	na	3	3	10	9	3	S<br>S<br>S	S<br>S<br>S	S<br>S<br>S	S<br>S<br>S	NO
**50**	950	3+	2	2+6	2+4	2	4	3	5	3	na	3	4	4	3+5+8	1	5	1	6	3	3	3	3	7	na	4	S<br>S<br>S	S<br>S<br>S	S<br>S<br>S	S<br>S<br>S	NO
**51**	891	4+	2	2	4	2	4	3	5	5	na	2	na	3	3+5+8	1	5	1	6	3	2	3	5	11	9	3	S<br>S<br>S	S<br>S<br>S	S<br>S<br>S	S<br>S<br>S	NO
**52**	354	3+	2	2	2	2	4	5	6	4	na	2	5	4	4+8	2	6	na	7+9	3	3	3	3	5	9	2+3	S<br>S<br>S	S<br>S<br>S	S<br>S<br>S	S<br>S<br>S	NO
**53**	3315	3+	2	2	2	2	5	5	5	4	na	2	5	4	3	2	5	1	9	3	2	3	3	10	na	3+4	S<br>S<br>S	S<br>S<br>S	S<br>S<br>S	S<br>S<br>S	NO
**54**	242	3+	2	2	3	2	4	4	5	na	na	6	4	na	3	na	6	1	5	3	3	3	na	na	9	3	S<br>S<br>S	S<br>S<br>S	S<br>S<br>S	S<br>S<br>S	NO
**55**	2784	3+	na	4	2	2	na	5	9	4	2	na	4	6	2	3	6	2	6	3	3	3	4	na	na	1	S<br>S<br>S	S<br>S<br>S	S<br>S<br>S	S<br>S<br>S	NO
**56**	1380	3+	na	2	4	0	na	1	8	5	na	2	3	5	1	1	5	1	6	2	2	3	3	na	na	na	S<br>S<br>S	S<br>S<br>S	R<br>R<br>R	S<br>S<br>S	NO
**57**	222	3+	2	2	4	3	3	2	3	na	2	na	3	5	2	3	6	2	4	4	4	3	na	3	2	2	S<br>S<br>S	S<br>S<br>S	S<br>S<br>S	S<br>S<br>S	NO

**58**	1647	NEG	2	4	3	3	na	na	na	na	2	na	na	na	8	na	7	4	na	na	4	3	na	na	na	na	S<br>S<br>S	S<br>S<br>S	R<br>R<br>R	S<br>S<br>S	NO
**59**	198	3+	2+11	6	na	3	1+3	3+7	3	0+2	na	na	5+8	3	1+4	2	4	1+2	4+5	1	1	3	3	7+8	3	1	S<br>S<br>S	S<br>S<br>S	S<br>S<br>S	S<br>S<br>S	NO
**60**	670	4AFB	na	na	na	3	1	na	na	na	na	na	na	na	1+4	na	4	na	na	na	na	3	na	na	na	na	S<br>S<br>S	S<br>S<br>S	S<br>S<br>S	S<br>S<br>S	NO
**61**	4551	3+	2	na	na	3	1+2	4	3	0	na	2	5+6	na	1+4	3	4	1	na	3	2	3	1+4	3	9	2	S<br>S<br>S	S<br>S<br>S	S<br>S<br>S	S<br>S<br>S	NO
**62**	4241	2+	2	6	10	3	1	4	2	na	5	3	3	4	1	3	6	1	3	3	2	3	3+6	5	2	1	S<br>S<br>S	R<br>R<br>R	S<br>S<br>S	S<br>S<br>S	NO
**63**	5039	NEG	2	na	na	3	1	4	na	na	na	na	3	4	1	3	na	1	na	3	2	3	na	na	na	na	S<br>S<br>S	R<br>R<br>R	S<br>S<br>S	S<br>S<br>S	NO
**64**	216	3+	2	4	4	3	1	5	2	2	na	9	5	4	4	3	5	1+2	1	3	1	3	3	8	3	2	S<br>S<br>S	S<br>S<br>S	S<br>S<br>S	S<br>S<br>S	NO
**65**	217	8AFB	2+3	na	na	3	1	na	na	1	na	na	na	na	1+4	3	na	1	na	3	na	3	3	4	8	3	S<br>S<br>S	S<br>S<br>S	S<br>S<br>S	S<br>S<br>S	NO
**66**	1479	NEG	na	na	na	3	na	9	2	na	na	na	na	na	6	11	na	na	na	na	4	3	na	na	7	na	R<br>R<br>R	R<br>R<br>R	R<br>R<br>R	S<br>S<br>S	NO

na: not amplified.

INH= Isoniazid; SM= Streptomycin; EMB = Ethambutol; RIF= Rifampicin; S= Susceptible; R= Resistant

All of the clinical samples were sputum (Except: Patient # 35 (Sample 2002: Pleural fluid); Patient# 40 (Sample 1871: Axillary abscess); Patient#53 (Sample 3315: Gastric juice); Patient# 49 (Sample 273: Gastric juice).
